# Sex and *APOE* genotype influence respiratory function under hypoxic and hypoxic-hypercapnic conditions

**DOI:** 10.1152/jn.00255.2023

**Published:** 2024-05-15

**Authors:** Chase E. Taylor, Laura E. Mendenhall, Michael D. Sunshine, Jessica N. Wilson, Chris M. Calulot, Ramon C. Sun, Lance A. Johnson, Warren J. Alilain

**Affiliations:** ^1^Department of Neuroscience, University of Kentucky, Lexington, Kentucky, United States; ^2^Spinal Cord and Brain Injury Research Center, University of Kentucky, Lexington, Kentucky, United States; ^3^Department of Biochemistry & Molecular Biology, College of Medicine, University of Florida, Gainesville, Florida, United States; ^4^Department of Biochemistry, University of Florida, Gainesville, Florida, United States; ^5^Center for Advanced Spatial Biomolecule Research, University of Florida, Gainesville, Florida, United States; ^6^Department of Physiology, University of Kentucky, Lexington, Kentucky, United States; ^7^Sanders-Brown Center on Aging, University of Kentucky, Lexington, Kentucky, United States

**Keywords:** APOE, breathing, hypercapnia, hypoxia, sex

## Abstract

The apolipoprotein E (*APOE*) gene has been studied due to its influence on Alzheimer’s disease (AD) development and work in an *APOE* mouse model recently demonstrated impaired respiratory motor plasticity following spinal cord injury (SCI). Individuals with AD often copresent with obstructive sleep apnea (OSA) characterized by cessations in breathing during sleep. Despite the prominence of *APOE* genotype and sex as factors in AD progression, little is known about the impact of these variables on respiratory control. Ventilation is tightly regulated across many systems, with respiratory rhythm formation occurring in the brainstem but modulated in response to chemoreception. Alterations within these modulatory systems may result in disruptions of appropriate respiratory control and ultimately, disease. Using mice expressing two different humanized *APOE* alleles, we characterized how sex and the presence of *APOE3* or *APOE4* influences ventilation during baseline breathing (normoxia) and during respiratory challenges. We show that sex and *APOE* genotype influence breathing during hypoxic challenge, which may have clinical implications in the context of AD and OSA. In addition, female mice, while responding robustly to hypoxia, were unable to recover to baseline respiratory levels, emphasizing sex differences in disordered breathing.

**NEW & NOTEWORTHY** This study is the first to use whole body plethysmography (WBP) to measure the impact of *APOE* alleles on breathing under normoxia and during adverse respiratory challenges in a targeted replacement Alzheimer’s model. Both sex and genotype were shown to affect breathing under normoxia, hypoxic challenge, and hypoxic-hypercapnic challenge. This work has important implications regarding the impact of genetics on respiratory control as well as applications pertaining to conditions of disordered breathing including sleep apnea and neurotrauma.

## Graphical abstract

**Figure:**
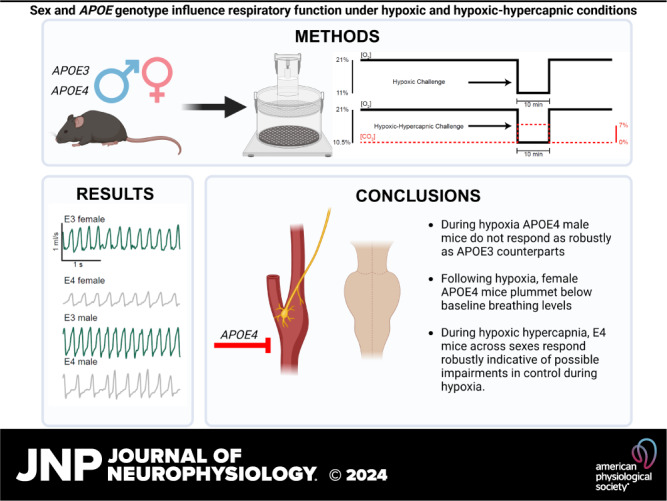

## INTRODUCTION

Apolipoprotein E (ApoE) has three distinct alleles: *APOE2*, -*E3*, and -*E4* each coding for a different lipid transport protein variant (1). The *APOE4* allele is a key genetic determinant for late-onset Alzheimer’s disease (AD); it is estimated that heterozygous carriage of the *APOE4* allele results in a fourfold increased risk of developing AD, whereas homozygous carriers exhibit a 15-fold increase (2). Approximately one in five people are carriers of the *APOE4* allele. Critically, in the context of our work, the *E4* Alzheimer’s connection has been linked to the presentation of cognitive decline and inflammation (3).

Breathing, the rhythmic inspiratory and expiratory patterns that facilitate gas exchange in the lungs, is essential to life, and conditions impacting respiratory function and neural respiratory control are critical in the context of aging-related disease (4–8). Obstructive sleep apnea (OSA) is a disease state that is characterized by cessations in breathing leading to periods of hypoxia (9). Repeated exposure to hypoxia leads to neuroinflammation and cognitive decline that can magnify the impacts of AD (10). Indeed, studies have shown that when conditions mimicking sleep apnea are used in animal models, inflammatory markers increase over time including markers of injury and neurodegeneration (11). Markers of inflammation and neurocognitive deficit have been readily observed across the human OSA population although some limited ischemic preconditioning may be responsible for the variability of outcomes observed (12). Although not well understood, numerous clinical studies have shown co-expression of cognitive decline or AD biomarkers in individuals with OSA (13–15).

Considering the importance of *APOE4* as the key genetic determinant for AD and hypoxia as a major driver of cognitive decline, it is critical to understand the impact and relationship of the *APOE4* allele on neural respiratory control and the body’s innate response to hypoxia, i.e., the hypoxic ventilatory response (HVR) (16). Although HVR has not been extensively studied in the context of OSA we believe that it may serve as an endogenous mechanism of overcoming the ill-effects of OSA and the presence of the *APOE4* allele may blunt this response. We tested the hypothesis that *APOE4* negatively impacts breathing relative to *APOE3* mice, particularly under conditions that would induce a response to respiratory stress. To test this hypothesis, we utilized whole body plethysmography (WBP) to measure ventilation in a humanized *APOE* mouse model of both sexes under normoxia (21% O_2_), and two different gas challenges (hypoxia alone and hypoxic-hypercapnia).

## METHODS

### Animals

All experiments were conducted in accordance with the NIH Guidelines Concerning the Care and Use of Laboratory Animals and were approved by the Institutional Animal Care and Use Committee at the University of Kentucky. Mice expressing human ApoE isoforms under control of the endogenous mouse *APOE* promotor (targeted replacement mice) were backcrossed for at least 10 generations to the C57BL/6 background (17, 18). Since deficits driven by *APOE4* become apparent later in life, aged mice were used (19). Mice were group-housed by sex and genotype on a 12/12 light/dark cycle and fed normal chow diet ad libitum (Teklad 2018 18% Protein Rodent Diet). Mice in *cohort 1* (hypoxic challenge; *n* = 33) weighed 36.9–48.6 g with ages ranging from 367–441 days. Mice in *cohort 2* (hypoxic-hypercapnic challenge; *n* = 16) weighed 32.02–52.7 g with ages ranging from 472–477 days. The average age and weight by sex, genotype, and cohort are listed in Table 1.

**Table 1.** Number, average weight, and average age of mice used in hypoxia and hypoxic-hypercapnia experiments

Cohort	Sex Age *P* < 0.01 Weight *P* < 0.01	Genotype Age *P* > 0.08 Weight *P* > 0.4	*n*	Average Weight, g (SD)	Average Age, days (SD)
Hypoxia challenge	Male	*E3*	9	48.9 (4.7)	410.6 (16.6)
		*E4*	8	45.5 (4.6)	441.3 (65.3)
	Female	*E3*	8	36.9 (7.5)	366.3 (10.1)
		*E4*	8	37.1 (5.4)	377.8 (8.1)

Cohort	Sex Age *P* > 0.18 Weight *P* <0.01	Genotype Age *P* > 0.8 Weight *P* < 0.01	*n*	Average Weight, g (SD)	Average Age, days (SD)
Hypoxic-hypercapnia challenge	Male	*E3*	4	52.7 (2.8)	473.0 (0)
		*E4*	4	47.5 (6.0)	473.5 (4.0)
	Female	*E3*	3	51.2 (5.9)	476.0 (0)
		*E4*	5	32.02 (4.2)	474.8 (3.8)

g, grams; SD, standard deviation.

### Hypoxia and Hypoxic-Hypercapnia Experiments

**Figure 1.**
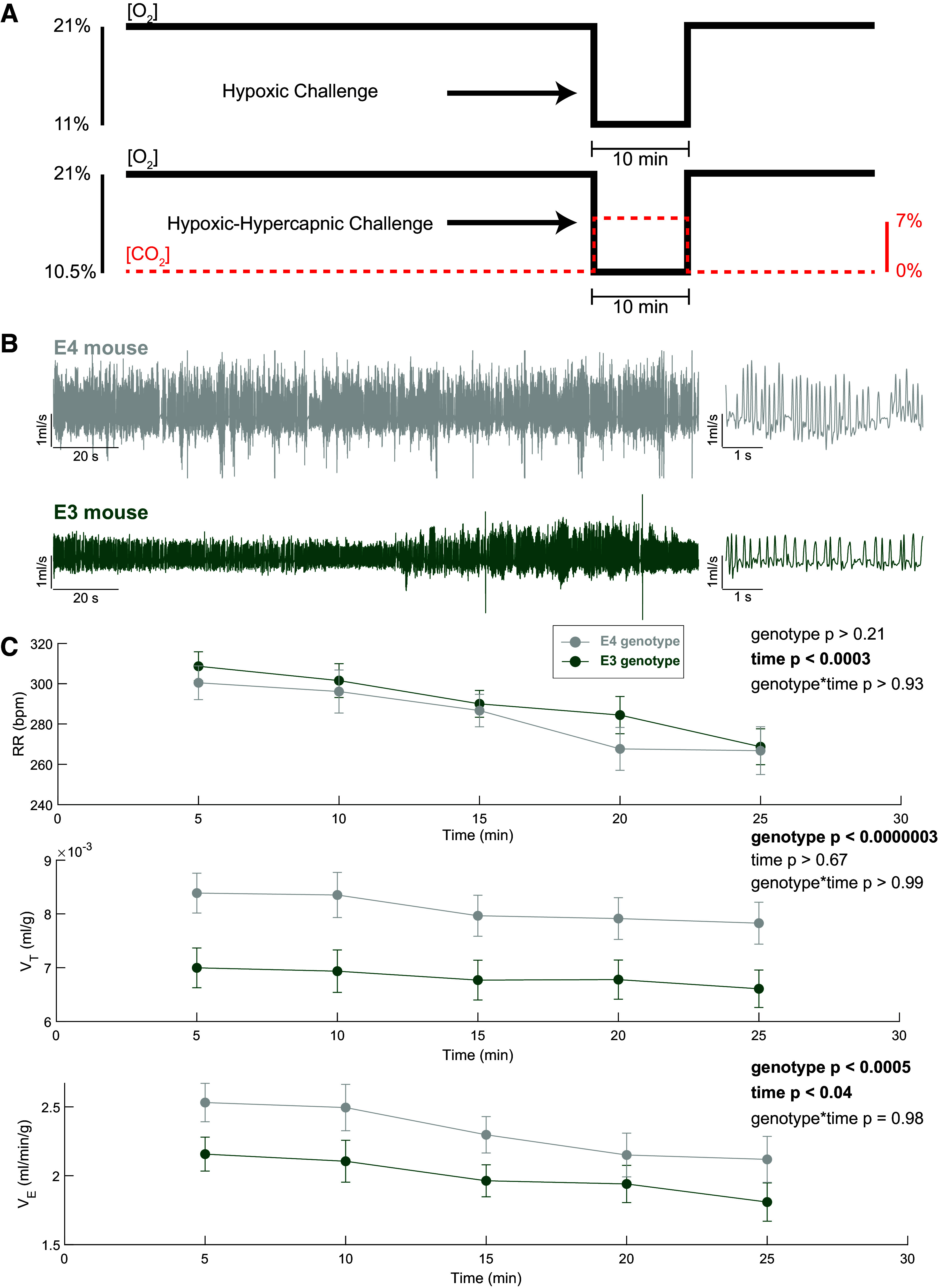
Apolipoprotein E (*APOE*)*4* animals across both sexes have increased tidal volume to maintain respiratory homeostasis under normoxic conditions while maintaining the same respiratory rate as *E3* mice. *A*: timeline of whole body plethysmography (WBP) hypoxic and hypoxic hypercapnic challenges. *B*: plethysmography airflow traces from a representative *APOE3* (green) or *APOE4* (gray) mouse during baseline (normoxia). *C*: quantification of plethysmography during baseline (normoxia) in 5-min bins, testing for an overall effect of genotype prior to assessing sex differences [two-way ANOVA means ± SE; *n* = 25 (*APOE4*, 13F/12M), *n* = 24 (*APOE3*, 11F/13M)]. RR, respiratory rate; V̇e, minute ventilation; Vt, tidal volume.

Whole body plethysmography (WBP, 1 L/min flow) (DSI, Buxco FinePointe) was used to measure the ventilatory response during hypoxic (11% O_2_ balance nitrogen) and hypoxic-hypercapnic (7% CO_2_, 10.5% O_2_, balance nitrogen) challenge in adult mice. Calibration was completed using DSI internal software included with the system. We calculated respiratory rate (breaths/min), tidal volume (mL/breath/g), and minute ventilation (mL/min/g) from the WBP flow traces. Whole body plethysmography recordings lasted approximately 90 min and occurred during daylight hours between 0930 and 1600 with food and water restricted during the 90-min measurement period. To ensure consistency across all measures, the WBP system was recalibrated prior to every trial. Immediately prior to baseline readings, a 40-min period of in-chamber acclimation occurred to reduce the prevalence of nonventilatory respiratory behaviors associated with exploring the chamber during the baseline period. Acclimation was followed by a 30-min period of normoxia (21% O_2_, balance nitrogen), then 10 min of respiratory challenge concluding with a 5- or 10-min period of normoxia. For the male mice in the hypoxic challenge group, only a 5-min postchallenge period was recorded. This period was later extended in our paradigm prior to collection of female mice to further elucidate postchallenge effects. The challenge design can be seen in Fig. 1*A* and the number of animals per group is shown in Table 1. Following trials, mice were returned to their home cage in the vivarium. WBP chambers were thoroughly cleaned and dried between each group.

### Data Analysis and Statistical Analysis

The normality and variance of the data were determined to ensure appropriate assumptions were met. If data within a particular experiment did not meet these assumptions nonparametric tests were used. Specific statistical tests for each figure are listed in the figure legends. Ventilatory measures were calculated in DSI FinePointe software and exported, breath by breath. The breath-by-breath data were used to calculate binned respiratory rate and average tidal volume, these metrics were multiplied to generate minute ventilation. This analysis and statistical testing was performed using MATLAB (MathWorks, Version 2021b). For hypoxic ventilatory challenge, hypoxic-hypercapnic ventilatory challenge, and postchallenge measures, data were normalized to percent change relative from baseline. A supplemental figure (Supplemental Fig. S1) was generated showing the progression of the hypoxic ventilatory response across all metrics in GraphPad (GraphPad Prism v.9.02). One-minute bins were used for Supplemental Fig. S1, for all other figures, 5-min bins were used. For statistical analysis, an ANOVA (or Kruskal–Wallis for nonparametric analyses) was used for statistical comparisons between sex and genotype. Tukey–Kramer post hoc test was used to test for differences within sex or genotype. Results were considered statistically significant if *P* < 0.05. Investigators were not blinded to genotype or sex.

## RESULTS

### Both Genotype and Sex Impact Normoxic Breathing with Female Mice Showing the Greatest Tidal Volume

**Figure 2.**
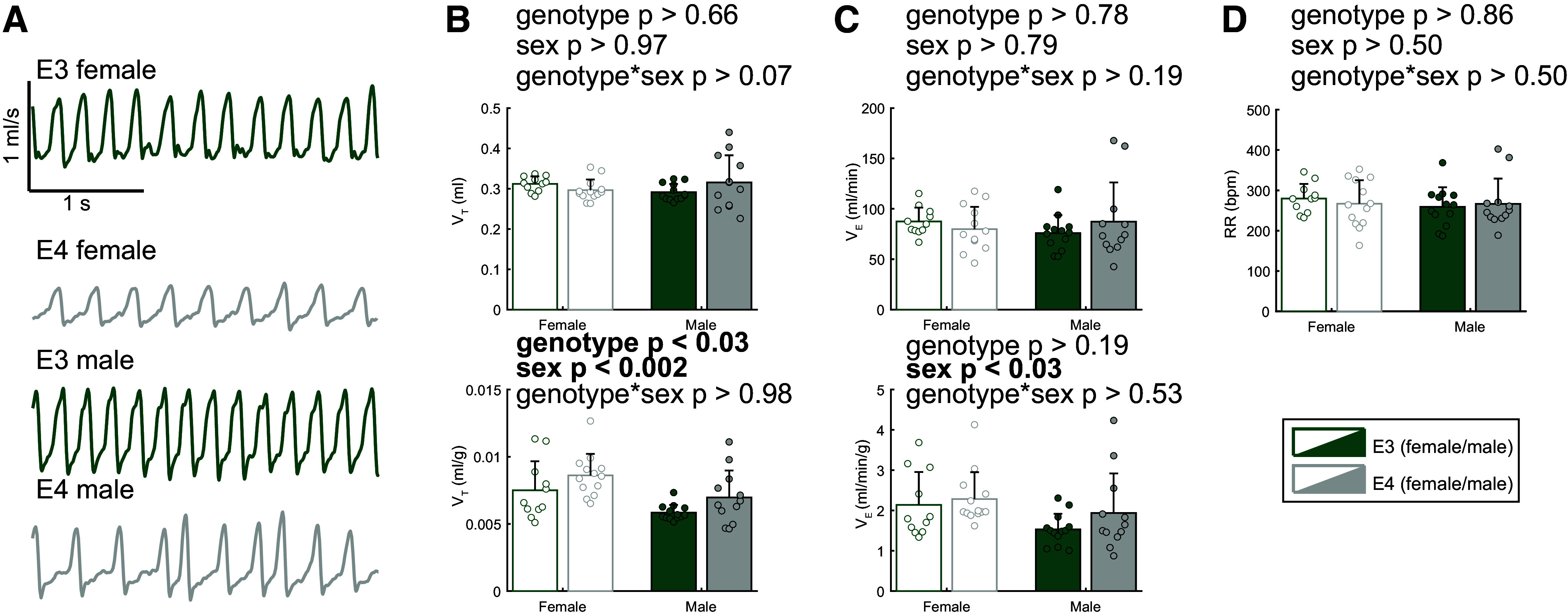
Female apolipoprotein E (*APOE*) mice breathe deeper than males to establish normoxic ventilatory patterns under stress-free conditions. *A*: representative airflow traces during baseline (normoxia) from *APOE3* and *APOE4* male and female mice. Metrics of ventilation at baseline tidal volume (Vt, mL) and weight-corrected tidal volume (Vt, mL/g) (*B*), minute ventilation (V̇e, mL/min) and weight-corrected minute ventilation (V̇e, mL/min/g) (*C*), and respiratory rate (RR (beats/min) (*D*). Male mice exhibit lower tidal volume and minute ventilation in weight-corrected values, and *APOE4* animals exhibit greater tidal volume than *E3* counterparts across sexes. Two-way ANOVA, means ± SD; *n* = 11 (*APOE3* female), *n* = 13 (*APOE4* female), *n* = 13 (*APOE3* male), *n* = 12 (*APOE4* male).

To determine differences in baseline breathing, *APOE3* and *APOE4* male and female mice underwent a 25-min period of normoxia prior to a 10-min respiratory challenge. Representative flow traces from an *APOE3* and -*E4* mouse (Fig. 1*B*) illustrate larger tidal volume during baseline (normoxia) in the *APOE4* mouse. Quantification of these data showed an effect of genotype with *E4* mice having a larger tidal volume throughout the normoxic baseline (Fig. 1*C*). Representative flow traces were shown from each sex and genotype with and without weight correction when applicable (Fig. 2*A*). Sex differences were present in tidal volume and minute ventilation when corrected for body weight (Fig. 2, *B–D*). Female mice had to breathe at a deeper and greater weight-corrected tidal volume relative to male mice to maintain baseline breathing (*P* < 0.002, *F*_1_ = 11.96). In addition, female mice had a larger weight-corrected minute ventilation (*P* < 0.03, *F*_1_ = 5.05). These baseline measures show a sex-dependent difference present that may affect how male and female mice will respond to a ventilatory challenge. As there was an effect of sex under baseline conditions, sex was then included as a primary variable in subsequent analyses. Finally, *E4* mice had larger weight-corrected tidal volume compared with *E3* mice (*P* < 0.03, *F*_1_ = 5.50).

### Male *APOE3* Mice Exhibit a Traditional HVR While Female Mice and *APOE4* Males Fail to Mount a Complete Response

**Figure 3.**
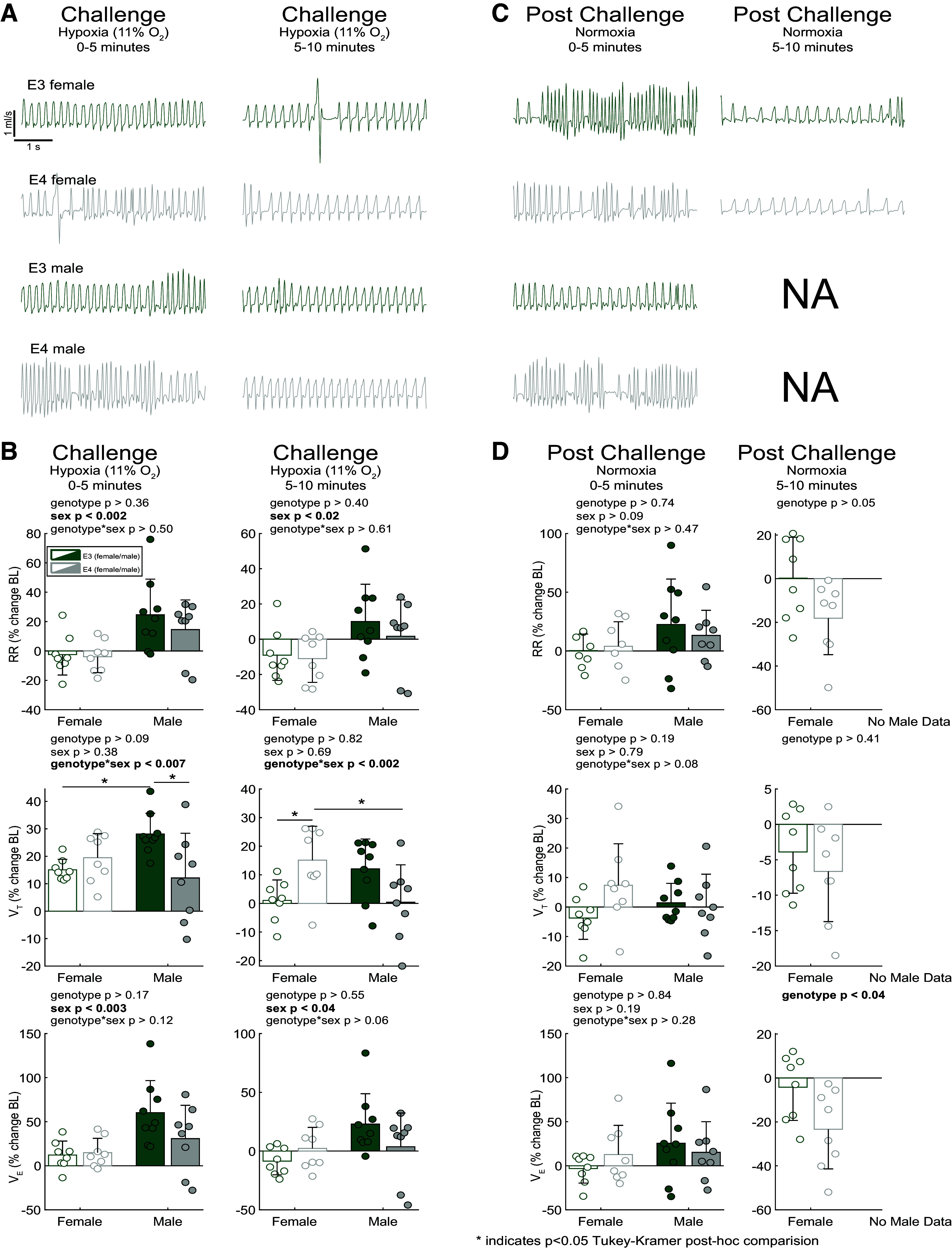
Male apolipoprotein E (*APOE*)*3* mice demonstrate a traditional hypoxic ventilatory response (HVR) stronger than *APOE4* males and appear to return to baseline levels following challenge whereas female mice fail to increase respiratory rate and *APOE4* females plummet to sub baseline levels following challenge. *A*: representative airflow traces during and after hypoxic ventilatory challenge. *B*: comparison graphs between sex and genotype during hypoxia. *C*: postchallenge airflow traces. *D*: ventilatory metrics during the postchallenge normoxic period. Initial studies conducted in male mice did not include 5–10 min postchallenge period. *P < 0.05, Tukey–Kramer post hoc. Two-way ANOVA, means ± SD; *n* = 8 (*APOE3* female), *n* = 8 (*APOE4* female), *n* = 9 (*APOE3* male), *n* = 8 (*APOE4* male).

The hypoxic ventilatory response is traditionally described by an increase in minute ventilation and tidal volume that peaks and is maintained for the duration of hypoxic challenge while frequency increases initially and is followed by a slight decrease prior to leveling out at a rate higher than baseline frequency (20). To investigate sex- and genotype-dependent respiratory differences, we exposed the animals to a 10-min hypoxic (11% O_2_) ventilatory challenge. Figure 3*A* shows airflow traces from animals undergoing hypoxic ventilatory challenge. Airflow data from all animals in the hypoxia cohort are quantified in Fig. 3*B*, showing the hypoxic challenge period, split into two 5-min bins. Within the first 5 min of hypoxia exposure, there is a clear effect of sex in respiratory rate and minute ventilation. During this initial period of hypoxia, when considering respiratory rate, male mice had an increase over female mice in their hypoxic response (*P* < 0.002, *F*_1_ = 12.82). During the final 5 min of the challenge, the respiratory rate of male mice returned toward baseline, whereas the respiratory rate of female mice declined further (*P* < 0.02, *F*_1_ = 6.57). All groups increased their tidal volume in the first 5 min of the hypoxic challenge. However, male *APOE4* mice had a less robust response compared with their *APOE3* counterparts (*P* < 0.05, Tukey–Kramer post hoc), supporting previous work showing that male *APOE4* mice have impaired ventilatory response under hypoxic conditions after spinal cord injury (SCI) (21). Furthermore, all groups increased their minute ventilation during the first 5 min of hypoxia. Only the *APOE3* male mice were able to maintain an elevated minute ventilation in the second half of hypoxia. Interestingly, female mice appeared to remain at or below their baseline minute ventilation during the latter portion of the challenge whereas *E4* male mice could not maintain tidal volume increases in response to hypoxia.

### Response and Recovery from Hypoxia Is Impaired in Female *APOE3* and *APOE4* Mice

Posthypoxia airflow traces appear to return toward baseline level (Fig. 3*C*). Following cessation of hypoxic challenge, there is a female genotype minute ventilation difference in the second 5-min postperiod (*P* < 0.04, Fig. 3*D*). When considering tidal volume and respiratory rate, female animals appear to show a decline relative to baseline, with greater deficit seen in *APOE4* female mice. Interestingly, there was a suggestion that female *E4* mice had an initial tidal volume increase relative to baseline following the hypoxic challenge followed by a plummet below baseline levels and the levels of female *E3* counterparts.

### During Hypoxic Hypercapnic Challenge *APOE4* Animals Are Able to Robustly Increase Respiratory Output Relative to *APOE3* Animals Regardless of Sex Although Sex Plays a Role during the Postchallenge Period

**Figure 4.**
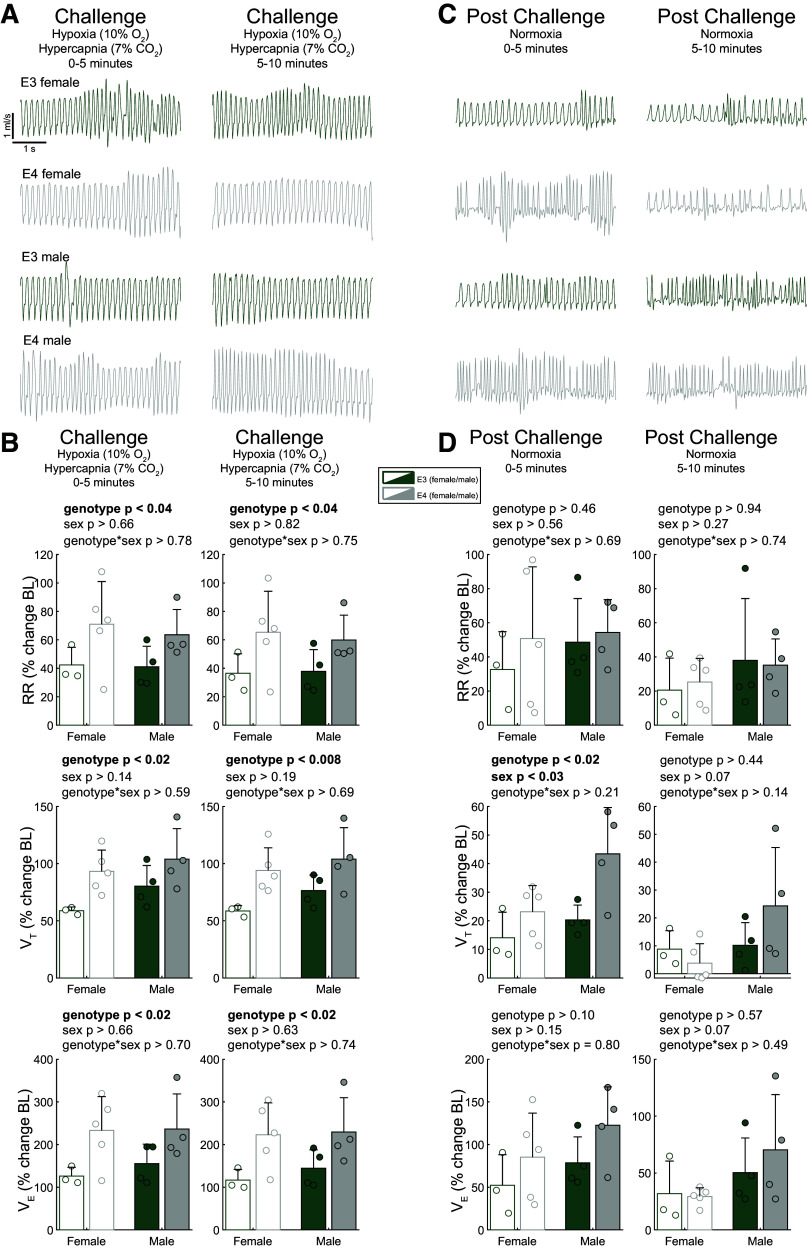
Under hypoxic hypercapnic conditions, apolipoprotein E (*APOE*)*4* animals respond robustly to challenge; however, *APOE4* males remain elevated relative to baseline following challenge. *A*: flow traces show a robust increase in tidal volume during challenge. *B*: comparison of ventilatory metrics during hypoxic-hypercapnic challenge, considering for effect of sex and genotype. *C*: representative airflow traces during post-hypoxic-hypercapnic ventilatory response (HHVR) challenge. *D*: respiratory metrics during the post-HHVR period. Two-way ANOVA, means ± SD; *n* = 3 (*APOE3* female), *n* = 5 (*APOE4* female), *n* = 4 (*APOE3* male), *n* = 4 (*APOE4* male).

As only the *APOE3* male group was able to produce and maintain a pronounced increase in minute ventilation during hypoxic challenge, we wanted to test if the other groups were limited in their ability to produce increased minute ventilation or if hypoxia alone simply did not trigger an increase. To accomplish this, in a separate cohort of animals, following the normoxic baseline period we challenged using a hypoxic-hypercapnic (10.5% O_2_, 7% CO_2_) mixture. As the male *APOE4* animals during hypoxia did not increase their tidal volume to the same degree as the male *APOE3* animals, we were specifically interested in the possibility of a male *APOE4* ceiling effect (i.e., inability to generate larger tidal volume). Figure 4*A* shows a robust hypoxic-hypercapnic ventilatory response (HHVR) across all mice, regardless of sex or genotype. This suggests that there was not a ceiling effect within the *APOE4* male mouse hypoxia cohort and increases across all measures could be solicited via a more severe respiratory challenge indicative of impaired chemoreception in male *APOE4* mice.

Figure 4*B* shows the entirety of the hypoxic-hypercapnic challenge and the corresponding response. The *APOE3* mice of both sexes had a lower respiratory rate compared with *APOE4* counterparts during the first 5 min (*P* < 0.04, *F*_1_ = 5.50) and the final 5 min (*P* < 0.04, *F*_1_ = 5.61). Minute ventilation mirrors this effect throughout hypoxic-hypercapnic challenge, driven by genotypic differences in both respiratory rate and tidal volume, indicating that *E3* mice have lowered respiratory capacity relative to *E4* mice during hypoxic-hypercapnic challenge.

Airflow traces from the period after the hypoxic-hypercapnic challenge (Fig. 4*C*) show a differential recovery based on sex and genotype. The male mice increased their respiratory rate in the first 5 min after the challenge, whereas the female mice maintained the same respiratory rate as during the challenge. The tidal volume of all *E3* mice and *E4* female mice appeared to return toward baseline, whereas the tidal volume of the *E4* male mice remained elevated.

The post-HHVR (Fig. 4*D*) male mice have a higher tidal volume than female mice across both genotypes in the first (*P* < 0.03, *F*_1_ = 6.93) 5 min with a trend in the second (*P* < 0.08, *F*_1_ = 3.88) 5 min. This data may be indicative of impaired respiratory control in the male mice, as they should return toward baseline levels following exposure to hypoxic-hypercapnic conditions.

## DISCUSSION

This study builds on previous research elucidating the effects of genetic influences and sex on respiratory control (21). Breathing is a tightly regulated system that incorporates both feedback and feedforward control; however, genetic disorders or predispositions can affect respiratory regulation (4–8). In prior work completed by our laboratory, female mice expressing the *E3* allele maintained diaphragmatic activity following intermittent hypoxia whereas *E4* females had significantly depressed activity (21). Unlike the female mice, males did not exhibit a difference in diaphragmatic activity based on genotype in our prior work. The results collected in this study continue to demonstrate that genotype and sex in humanized *APOE* mice play a role in regulating breathing, specifically under conditions of respiratory stress. A summary of the individual changes can be referenced in Table 2.

**Table 2.** Summary of significant results across baseline measures and challenges

Normoxia	Baseline Measures			
Sex effects	• Females have higher weight-corrected tidal volume • Females have higher weight-corrected minute ventilation.			
Genotype effects	• E4 animals have higher weight-corrected tidal volume.			
Interaction effects	• None			

Hypoxia	First 5 Min	Second 5 Min	Posthypoxia First 5 Min	Posthypoxia Second 5 Min
Sex effects	• Males have increased respiratory rate • Males have increased minute ventilation	• Males have increased respiratory rate • Males have increased minute ventilation	• None	• None
Genotype effects	• None	• None	• None	• E4 females have depressed minute ventilation relative to *E3* females
Interaction effects	• *E3* Males have increased tidal volume compared to *E3* females and *E4* males	• *E4* females have increased tidal volume compared to *E3* females and *E4* males	• None	• None

Hypoxic-Hypercapnia	First 5 Min	Second 5 Min	Posthypoxia-Hypercapnia First 5 Min	Posthypoxia-Hypercapnia Second 5 Min
Sex effects	• None	• None	• Males haveincreased tidalvolume	• None
Genotype effects	• *E4* animals have increased tidal volume, respiratory rate and minute ventilation	• *E4* animals have increased tidal volume, respiratory rate and minute ventilation	• *E4* animals have increased tidal volume	• None
Interaction effects	• None	• None	• None	• None

### Genotype and Sex Differences Seen during Hypoxia Are Reversed or Eliminated When Hypercapnia Is Introduced

Obstructive sleep apnea results in periods of low tissue oxygenation that co-occur with retention of CO_2_ (22). These periods of low oxygen and high carbon dioxide are associated with increased neuroinflammation and impaired cognition (23–25). Low levels of oxygen within the brain results in ischemia-reperfusion events. In addition, the reoxygenation period appears to be a driver of the proinflammatory process (26). Many factors contribute to the occurrence of both obstructive and central apnea including age, sex, and genetic determinants (27). Indeed, congenital central hypoventilation syndrome, which is the result of a genetic mutation, leads to fatal apneas because of impaired CO_2_ sensing (28, 29). It follows that genetic polymorphisms such as *APOE* may play a critical role in the onset and severity of OSA pathology and symptoms (30, 31).

Although *APOE* genotype has been evaluated within sleep disordered breathing across broad datasets in human subjects, the research investigating animal models of *APOE* under respiratory stress has been limited (32, 33). We aimed to tease apart how *APOE* genotype impacts ventilation during hypoxia or hypoxic-hypercapnia that model mild and more severe apneas, respectively.

When exposed to hypoxia, our *APOE4* male mice initially increased respiratory rate and tidal volume, but were unable to maintain this increase in frequency or tidal volume in response to this stimulus. These findings suggest that male *APOE4* carriers have a reduced drive to breathe that may elongate apneas. However, in the presence of a combined hypoxic-hypercapnic challenge that models more severe apneas, the *APOE4* male mice were able to produce and maintain a large increase in minute ventilation driven by both respiratory rate and tidal volume. Although the *APOE4* female mice were able to increase and maintain tidal volume, upon completion of the challenge they experience a large drop in minute ventilation apparently below their baseline minute ventilation and certainly relative to the *E3* female mice. These changes indicate differences in respiratory responsiveness and suggest reduced plasticity may be an eventual outcome that would result in an inability of *APOE4* carriers across sexes to adapt to periods of hypoxia during OSA (34–36). Indeed, the *E4* male carrier hypoxia response deficit may actually limit their ability to develop one possible compensatory mechanism of plasticity over longer durations of hypoxia to overcome OSA impairments known as respiratory-long term facilitation (LTF) (37). This same interpretation can be applied to female *APOE4* mice who generate a robust HVR but show a deficit in breathing relative to baseline normoxia following the challenge. Indeed, in human studies, recent work by Nair et al. (33) has shown that the APOE4 allele negatively impacts respiratory motor plasticity in humans relative to APOE3.

Although limited research asserts that *APOE4* may not contribute to OSA, our research has continued to show that the ability to attenuate a hypoxic insult such as that seen in OSA is linked to the *APOE4* genotype (38, 39). As stated before, hypoxia and the inability to compensate for it has been clearly shown to drive neuroinflammation and cognitive impairment. Chemoreceptor function under hypoxia is susceptible to genetic alterations demonstrated in carotid bodies, although models of humanized *APOE* mice have not yet been used (40).

Interestingly, our hypoxic-hypercapnic ventilatory challenge data show that *E4* animals can indeed increase their respiratory output in a stimulus-dependent manner. During hypoxic-hypercapnic challenge, tidal volume and minute ventilation increased 50–200% relative to baseline. We show this across both genotypes and sexes with *E4* animals actually responding more robustly relative to *E3* counterparts. This is particularly important since it shows that *APOE4* animals have the ability to increase respiratory drive but not under conditions of hypoxia such as one would primarily experience during OSA. One reason for this may be that hypercapnic chemoreception is elicited primarily centrally in the retrotrapezoid nucleus (RTN) whereas hypoxic chemoreception takes place peripherally in the carotid and aortic bodies (41–43). It could therefore be surmised that while mechanisms of peripheral chemoreception may be impaired in *E4* animals, central hypercapnic chemoreception remains unaffected or highly responsive to changes in Pco_2_ content. Since respiratory drive remains intact, pathways leaving brainstem respiratory centers may also be unaffected by *APOE*. These findings suggest that the elicitation of Pco_2_ chemoreceptive drive may represent one possible intervention for OSA individuals heterozygous or homozygous for *APOE4*. It is worth noting that hypercapnia is also a feature of obstructive sleep apnea and there is cross talk between the sites of central and peripheral chemoreception and these interactions should be further studied in our model (44).

Sex-dependent responses to hypoxia and hypoxic-hypercapnia have been studied in other animals, although the most relevant background to our current findings was a study in Fischer rats showing that ventilatory response differences are age-dependent as opposed to sex-dependent with middle aged and older female rats having a greater response (45, 46). Interestingly, in a study determining central chemoreceptor function, sex differences in mice were demonstrated with male mice displaying a much higher number of RTN cells stimulated following exposure to hypercapnia (46). Sex differences in respiratory recovery have also been noted previously in neonatal mouse models where female respiratory rhythm generating centers have shown greater ability to recover following a period of severe hypoxia (47). Interestingly, in one study of wild-type C57BL6 mice, animals of both sexes displayed a strong HVR, the limited response of our APOE3 female mice contradicts these findings and may point to an effect of genotype on breathing mediated by the APOE3 allele (48). One additional consideration, mice have been known to decrease metabolic rate in response to hypoxic conditions leading to lower CO_2_ production, however, we have no reason to believe at this time that the reduction in metabolic rate would not be uniform across genotype. Future studies will further elucidate this potential confound by measuring blood gases (49).

Future research will elucidate *APOE-*specific differences in phrenic motor neuron serotonergic signaling (21). In addition, while inclusion of wild-type animals are not normally included in studies comparing human APOE genotype, future work might benefit by including a cohort of aged-matched wild-type male and female controls (1, 18). The data presented here serve to fill a critical gap in knowledge as these *APOE* mice are both closer in relative age and more closely model conditions akin to human sleep apnea physiology (50).

### Conclusions

This study further clarifies work from our laboratory and others on the effects of *APOE* on respiratory control in unfavorable respiratory conditions. Our results show that humanized *APOE4* male mice have more difficulty mounting a response to hypoxic conditions than their *E3* counterparts whereas female mice of both genotypes appear to have difficulty mounting any response in terms of respiratory rate and only *E4* females maintain a respective tidal volume increase. Interestingly, *E4* female mice have prolonged respiratory depression following cessation of hypoxia, not seen in their male counterparts (16).

In hypoxic-hypercapnic conditions, genotype differences persist across all measures, however, more interesting perhaps are the sex differences seen in recovery following hypoxic-hypercapnia. Because sleep apnea and disordered breathing have been indicated in earlier onset of Alzheimer’s disease, as well as cognitive impairment, this work will serve as a foundation for researching downstream effects of hypoxia and hypoxic-hypercapnia in APOE genotype and sex. Moreover, these findings may be of importance toward studying the impact of the genetically heterogenous human population and its relevance to disease and neurotrauma (21, 51–55).

## DATA AVAILABILITY

The datasets and MATLAB code generated during the current study are available from the corresponding author on reasonable request.

## SUPPLEMENTAL DATA

10.5281/zenodo.10957287 Supplemental Fig. S1: https://doi.org/10.5281/zenodo.10957287.

## GRANTS

This study was supported by funding the Craig H. Neilsen Foundation (to W.J.A.) and the Kentucky Spinal Cord and Head Injury Research Trust (to W.J.A.). Additional funding sources include NIH RO1s: AG065220 (to L.A.J.), AG060056 (to L.A.J.), AG80589 (to L.A.J.), AG081421 (to L.A.J.), AG066653 (to R.C.S.), CA266004 (to R.C.S.), AG078702 (to R.C.S.), R01CA288696 (to R.C.S.), RM1NS133593 (to R.C.S.); the Alzheimer’s Association (to L.A.J.), and the V-Scholar Grant (to R.C.S.).

## DISCLOSURES

No conflicts of interest, financial or otherwise, are declared by the authors.

## AUTHOR CONTRIBUTIONS

C.E.T. and W.J.A. conceived and designed research; C.E.T. and L.E.M. performed experiments; C.E.T., L.E.M., and M.D.S. analyzed data; C.E.T., L.E.M., M.D.S., J.N.W., C.M.C., R.C.S., L.A.J., and W.J.A. interpreted results of experiments; C.E.T., M.D.S., and W.J.A. prepared figures; C.E.T., L.E.M., M.D.S., and W.J.A. drafted manuscript; C.E.T., L.E.M., M.D.S., J.N.W., C.M.C., R.C.S., L.A.J., and W.J.A. edited and revised manuscript; W.J.A. approved final version of manuscript.
